# A Rare Association of Epstein-Barr Virus and Budd-Chiari Syndrome

**DOI:** 10.7759/cureus.52323

**Published:** 2024-01-15

**Authors:** Eli A Zaher, Mohamed A Ebrahim, Parth Patel, Shayet Hossain Eshan, Muhammad Sohaib Alvi

**Affiliations:** 1 Internal Medicine, Ascension Saint Joseph Hospital, Chicago, USA

**Keywords:** antiphospholipid antibody syndrome (aps), mrcp imaging, thrombosis, budd-chiari syndrome, epstein barr virus (ebv)

## Abstract

Budd-Chiari syndrome (BCS) is a rare hepatic venous outflow obstruction typically associated with hypercoagulable states. We present a unique case of a 29-year-old male with BCS triggered by a recent Epstein-Barr virus (EBV) infection. Workup unveiled antiphospholipid antibody syndrome as an underlying prothrombotic condition. Diagnostic challenges included inconclusive ultrasound findings, necessitating magnetic resonance imaging for confirmation. This case underscores the importance of considering infectious triggers for venous thromboembolism in BCS. Understanding the potential link between EBV and thrombosis warrants further investigation.

## Introduction

Budd-Chiari syndrome (BCS) is a rare form of congestive hepatopathy caused by obstruction to the hepatic venous outflow. Blockage of at least two hepatic veins is required for clinical disease, which may present as abdominal pain, ascites, hepatomegaly, and the sequelae of portal hypertension in chronic cases. At least one hypercoagulable condition is identified in 80% of cases, usually in combination with a triggering factor [1]. To our knowledge, this is the second case depicting Epstein-Barr virus (EBV) as a potential trigger for BCS in a predisposed individual [2]. 

## Case presentation

A 29-year-old immunocompetent male presented to the emergency department with complaints of bloating and progressive abdominal distension for the preceding one month. He additionally reported nausea with reduced appetite, low-grade fever, fatigue, sore throat, shortness of breath, and occasional watery diarrhea. History was negative for jaundice, change in stool color, substance or alcohol use, traveling, or exposure to sick contacts or pets. He did not have any significant past medical history and was not on any chronic medications. His family history was unknown given that he was adopted. 

On examination, his abdomen was distended without tenderness or guarding. Eye examination demonstrated scleral icterus. The lungs were clear to auscultation, and his skin examination was normal. Admission vitals showed tachycardia with 120 bpm but were otherwise normal. Initial significant laboratory workup showed a total bilirubin of 3.5 mg/dl (reference range 0-1.0 mg/dL), direct bilirubin of 1.32 mg/dL (reference range 0-0.2 mg/dL), aspartate aminotransferase of 56 IU/L (reference range 13-39 IU/L), alanine aminotransferase of 38 IU/L (reference range 7-52 IU/L), alkaline phosphatase of 78 (reference range 40-129 IU/L), albumin of 3.4 g/dL (reference range 3.5-5.7 g/dL), creatinine of 0.92 mg/dL (reference range 0.7-1.3 mg/dL), sodium of 133 mmol/L (reference range 133-144 mmol/L), platelet count of 124 cells/µL (reference range 150-450 cells/µL), and INR of 3.1 (reference range 0.9-1.1). Both the hepatitis and human immunodeficiency virus panels were negative. Ferritin and ceruloplasmin were within normal limits. Antinuclear, antimitochondrial, and antismooth muscle antibodies were negative. The MELD-Na score was 28. 

He underwent ultrasound-guided paracentesis with a total of 1720 mL of yellow ascitic fluid removed. Peritoneal fluid analysis demonstrated an albumin of 1.7 g/dL and a SAAG of 1.7, consistent with portal hypertension. Additional analysis showed a cell count of 47 cells/µL (reference range <250 cells/µL), an amylase of <10, triglycerides of 45 mg/dL (reference range <65 mg/dL), and an LDH 98 U/L (reference range <68 U/L). Microbiologic workup of ascitic fluid including acid-fast and AFB smear, gram stain, and culture was negative. 

**Figure 1:**
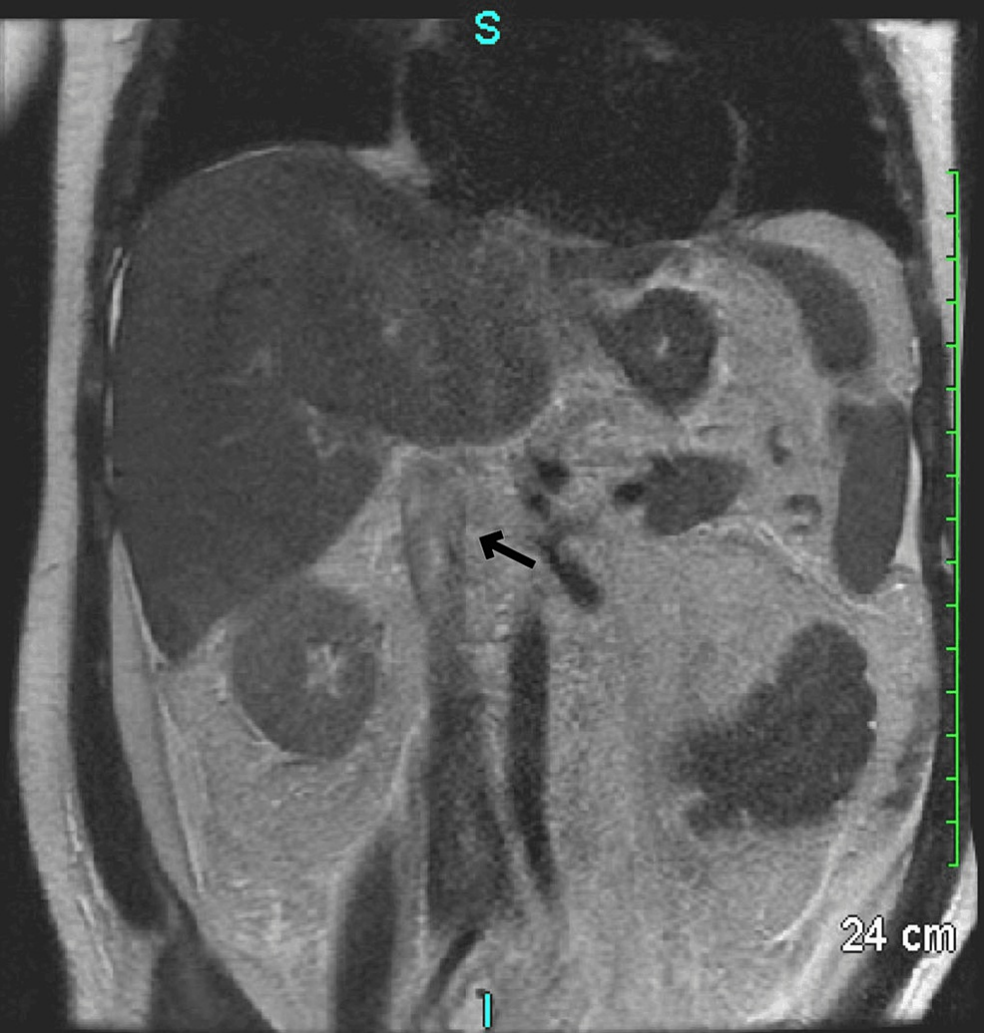
MRI Abdomen Arrow pointing toward a filling defect in the inferior vena cava.

Given the evidence of portal hypertension from the ascitic fluid analysis, an abdominal ultrasound with Doppler was done without evidence of portal vein or inferior vena cava (IVC) thrombosis. The hepatic echotexture was moderately echogenic, suggestive of diffuse fatty infiltration. This was followed by an MRI of the abdomen which was consistent with BCS (Figure 1). 

Hypercoagulability workup was thus initiated and demonstrated positivity for anti-beta-2 glycoprotein-I which was reconfirmed as positive 12 months later, establishing the diagnosis of antiphospholipid antibody syndrome. 

Considering our patient’s chronic sore throat, an infectious disease panel was sent and came back positive for EBV. The EBV panel was consistent with a recent infection as his viral capsid antigen (VCA) IgM, VCA IgG, and early antigen antibody were positive and the nuclear antigen antibody was negative. 

Our patient was thus suspected to have had a thrombotic predisposition with antiphospholipid antibody syndrome that in combination with EBV infection led to IVC thrombosis. He was transferred to a tertiary care center and successfully underwent IVC recanalization with balloon angioplasty and was discharged home on warfarin.

## Discussion

BCS is a rare condition marked by the blockage of hepatic venous outflow, which can occur due to thrombotic or non-thrombotic obstructions at any point from the hepatic venules to the junction of the IVC and the right atrium. BCS has an incidence of 1 in 100,000 of the general population [3]. BCS presents as a diverse clinical entity, with outcomes ranging from curable to potentially fatal. Proper management offers patients a favorable prognosis compared to other chronic liver diseases. This syndrome holds significance due to its rarity and the potential for complicating various disorders, such as hematologic or malignant conditions [4].

Diagnosis requires a high index of suspicion and radiologic assistance. Doppler ultrasonography is the first-line modality although it can miss about 15% of cases, just as happened with our patient [1]. Ultrasonography likewise cannot evaluate the extrahepatic collateral pathways, posing a significant limitation. MRI has the highest sensitivity for the detection of BCS; however, it can be limited in those with massive ascites due to artifacts and difficulty with laying down for the scan [5]. 

Thrombosis is the major cause of hepatic vein obstruction. In 80% of patients, at least one hypercoagulable state, whether hereditary or acquired, could be identified, while 20% of patients might have multiple contributing factors. Additionally, a predisposing disorder might manifest after the diagnosis of BCS [6]. There's a growing body of evidence indicating that infections can trigger venous thromboembolism (VTE). Various theories have outlined the connection between viral infections, the coagulation pathway, and the hemostasis system. These theories primarily revolve around endothelial inflammation and damage resulting from uncontrolled viral replication [7].

EBV infection commonly presents with either no symptoms or flu-like manifestations. However, in some cases, it can lead to conditions such as mononucleosis syndrome, which mainly presents with hepatosplenomegaly, cervical lymphadenopathy, pharyngitis, hepatitis, monocytosis, and atypical lymphocytosis [8]. While there are multiple instances documented in medical literature linking cytomegalovirus to VTE, reports of EBV causing VTE are rarer to observe as noted in this case report [9]. The complete understanding of how EBV infection initiates thrombosis remains unclear. Possible contributing factors could involve temporary increases in antiphospholipid antibodies and oxidative damage to endothelial cells induced by EBV [10,11].

## Conclusions

BCS is a rare form of congestive hepatopathy typically occurring in predisposed individuals in the setting of a prothrombotic trigger. Our case illuminates the possible role of EB infection acting as a trigger to BCS. Although the inflammatory condition itself might have heightened the risk of VTE, it is essential not to overlook the potential for any other mechanisms contributing to VTE in this case. Consequently, we aim to highlight EBV infection as a potential triggering factor for VTE. Further studies are needed to investigate the occurrence and correlation between EBV and VTE.
